# Mental health and loneliness in Scottish schools: A multilevel analysis of data from the health behaviour in school‐aged children study

**DOI:** 10.1111/bjep.12581

**Published:** 2023-01-23

**Authors:** Claire Goodfellow, Malachi Willis, Joanna Inchley, Kalpa Kharicha, Alastair H. Leyland, Pamela Qualter, Sharon Simpson, Emily Long

**Affiliations:** ^1^ MRC/CSO Social and Public Health Sciences Unit University of Glasgow Glasgow UK; ^2^ Campaign to End Loneliness part of What Works Centre for Wellbeing London UK; ^3^ Manchester Institute of Education University of Manchester Manchester UK

**Keywords:** adolescence, loneliness, multilevel modelling, school mental health

## Abstract

**Background:**

Adolescent loneliness and poor mental health represent dual public health concerns. Yet, associations between loneliness and mental health, and critically, how these associations vary in school settings are less understood.

**Aims:**

Framed by social‐ecological theory, we aimed to identify key predictors of adolescent mental health and examine school‐level variation in the relationship between loneliness and mental health.

**Sample:**

Cross‐sectional data on adolescents from the 2018 wave of the Health Behaviour in School‐aged Children study (HBSC) in Scotland were used (*N* = 5286).

**Methods:**

Mental health was measured as a composite variable containing items assessing nervousness, irritability, sleep difficulties and feeling low. Loneliness was measured via a single item assessing how often adolescents felt ‘left out’. Multilevel models were used to identify social‐ecological predictors of mental health, associations with loneliness and between‐school variation.

**Results:**

Loneliness, as well as demographic, social and school factors, was found to be associated with mental health. Mental health varied across schools, with the between‐school difference greater among adolescents with high levels of loneliness. Additionally, the negative effect of loneliness on mental health was stronger in schools with lower average mental health scores.

**Conclusions:**

The findings suggest that schools can play an important role in shaping adolescent mental health. Our study uniquely identifies that school‐based interventions targeting mental health may be especially necessary among lonely adolescents, and programmes aimed at tackling loneliness may be more beneficial in schools with poorer mental health.

## INTRODUCTION

Loneliness represents a significant public health concern due to its negative impacts on physical and mental health (Goosby et al., 2013; Qualter et al., 2013). Loneliness is especially prevalent among young people, with those aged 16–24 years most likely to experience loneliness (Office for National Statistics; ONS, 2018a). Despite this, most of the research and policy attention on loneliness has overlooked young people (What Works Centre for Wellbeing, 2019). Therefore, less is known about the role of loneliness among adolescents under the age of 16 years and, specifically, associations between loneliness and mental health in this age group.

Adolescence is a particularly vulnerable period for the development of mental health problems, with half of all mental health problems emerging before the age of 14 (Kessler et al., 2007; World Health Organization, 2018). Moreover, international evidence indicates rates of mental health problems among adolescents are increasing (Bor et al., 2014; Collishaw, 2015). This rise in poor youth mental health is notably steeper in the United Kingdom (Inchley et al., 2020; UNICEF, 2020), and particularly in Scotland (Currie et al., 2015; SAMH, 2018) when compared to other high‐income countries. Additionally, poor adolescent mental health is known to persist into adulthood (Shore et al., 2018), indicating a pressing need to identify modifiable factors associated with poor mental health during this developmental period.

### Youth loneliness and mental health

Loneliness is the discrepancy between desired and perceived social relationships (Perlman & Peplau, 1981), representing a painful and subjective experience which draws on an individual's expectation and satisfaction with the frequency and closeness of their contacts (de Jong Gierveld & Havens, 2004).

Adolescents are at heightened risk of loneliness due to developmental shifts in social networks, where the primary source of socialization shifts from parents towards peers (Goossens, 2018; Laursen & Hartl, 2013). Adolescence is also marked by periods of transition (e.g., moving from primary to secondary school, or leaving home), which is a known risk factor for increased loneliness (Siva, 2020; Sundqvist & Hemberg, 2021).

While the predominant body of research on the relationship between loneliness and mental health in based on older adult populations (Holt‐Lunstad et al., 2015; Public Health England, 2015), we do know that loneliness in adolescence is a risk factor for anxiety (Maes et al., 2019), depression (Fontaine et al., 2009; Lasgaard et al., 2011), suicidal ideation (Gallagher et al., 2014) and diminished positive mental health (Lyyra et al., 2021). We also know that, while the use of social media among adolescents is pervasive (Boer et al., 2020), frequent use of social media may in some cases be associated with increased loneliness (Azhari et al., 2022; Smith et al., 2021). However, while the associations between adolescent loneliness and mental health are beginning to emerge in the literature, a focus on how loneliness relates to mental health within school settings is currently lacking.

### Mental health and loneliness in schools

The school environment is closely linked with adolescent mental health and well‐being (Aldridge & McChesney, 2018). For example, poor school climate (i.e., the quality and character of the school environment, including norms and values, Gage et al., 2014) is linked with increased risk of poor mental health among students (László et al., 2019; Long et al., 2020). Schools that place emphasis on academic achievement and examination performance have been found to be associated with poorer mental health (Byrne et al., 2007; Högberg et al., 2020), while a supportive teacher relationship is associated with better mental health (Miller‐Lewis et al., 2014; Wang et al., 2013). Moreover, school disengagement is a key contributing factor to worsening mental health among adolescents (Sweeting et al., 2010), while increased school attachment can be a protective factor for adolescent mental health (Giordano, 2003). As such, adolescents' experiences at school are a vital component of their mental health and well‐being. Loneliness is also influential in adolescents' mental health. In a school context, loneliness plays an important role in mediating school belongingness and subjective well‐being (Arslan, 2021) and is higher among adolescents who feel less integrated into their school (Chipuer, 2001; Kingery & Erdley, 2007). Additionally, an increased sense of school connectedness can reduce loneliness and buffer mental health among adolescents (Benner et al., 2017; Cavanaugh & Buehler, 2016). Young people have suggested numerous preventative measures, including increased education relating to loneliness, support from teachers and learning to cope with emotions (Sundqvist & Hemberg, 2021). Indeed, such is the importance of schools for loneliness and mental health that young people themselves have identified schools as playing a vital role in tackling loneliness and, by doing so, protecting their mental health (Mental Health Foundation, 2021). As such, it is crucial that research considers loneliness and mental health together in school settings. A clearer understanding of the extent to which loneliness relates to mental health, and particularly whether this relationship is consistent across different schools, would offer new insight into the types of settings where whole‐school interventions targeting loneliness are likely to have the most significant impact on adolescents' mental health.

### Social‐ecological framework

From a social‐ecological perspective (Bronfenbrenner, 2005; Sallis et al., 2008), adolescent mental health is shaped by multiple, interacting layers of influence at the micro (individual) meso (social relationships) and macro (community and environment) level. For adolescents, these layers of influence include demographic factors, peer and family relationships, and the wider community or school context. Within this framework, the current study investigates correlates of adolescent mental health across a range of demographic, social and school factors within adolescents' lives, while accounting for school‐level differences in mental health. Importantly, while individual‐level demographic factors may highlight particular risks for poor mental health, these may be unmalleable (e.g., gender). However, social and school factors may be more easily integrated into school‐based interventions.

While previous research has utilized multilevel statistical analyses to determine whether mental health varies significantly between schools, with mixed findings (Levin et al., 2012; Long et al., 2020; Patalay et al., 2020), to the best of our knowledge, no other research has explored between‐school differences in the relationship between adolescent loneliness and mental health. Therefore, this study furthers current evidence by examining whether the effect of loneliness on mental health is stronger in some schools than others. This will allow for the development of targeted public health or whole‐school interventions to improve mental health.

### Study aims

The aims of the study were to (a) identify key social‐ecological predictors of mental health, (b) assess the extent to which loneliness is related to mental health among adolescents and (c) determine whether there is between‐school variation in the association between loneliness and mental health.

Specifically, this study aimed to answer the following research questions:

1. What are the key social‐ecological predictors of adolescent mental health?
2. Is greater loneliness associated with poorer mental health among adolescents, after adjusting for social‐ecological predictors?
3. Does the relationship between loneliness and mental health differ according to the school adolescents attend?

## MATERIALS AND METHODS

### Data and participants

Data were collected as part of the 2017/18 Health Behaviour in School‐aged Children (HBSC) study in Scotland. The HBSC is a World Health Organization Collaborative Cross‐National Survey, which runs every 4 years. All countries participating in the study follow a standardized protocol. The survey is administered in schools to 11‐, 13‐ and 15‐year‐olds and collects data on health and health behaviours, as well as a range of school and social factors. Our sample included 5286 participants, with a mean age of 13.56 years. The sample was 51.3% female. To minimize selection bias, the HBSC study uses a proportionally stratified sample, which is stratified by school funding (state or independent) and by local education authority, as well as other factors to ensure that each pupil within a stratum has the same probability of inclusion in the sample.

### Materials and measures

Framed by social‐ecological theory, our study investigated predictors of adolescent mental health across three domains: individual factors, social‐relationship factors and community factors, while controlling for school‐level clustering. A full list of variables in each domain is included below, and in Table 1. Missingness of variables ranged from 0 to 8.76% (see Table 1).

**TABLE 1:** Descriptive statistics

Domain	Variable info	Mean	*SD*	Scale info	Missingness
Outcome	Mental Health	14.68	4.27	1–20 (higher score = better mental health)	2.10%
Individual / demographic	Left out	2.40	.93	1 (never) – 5 (always)	.66%
	Gender	1.51	.50	51.35% female; 48.99% male	0
	Age	13.56	1.65	8.3 years – 18.6 years	3.37%
	SES	8.98	2.38	1 (low SES) – 13 (high SES)	4.39%
	Health	3.03	.67	1(poor health) – 4 (excellent health)	.70%
	Life satisfaction	7.61	1.90	1 (worst) – 10 (best)	.64%
Social relationships	Family communication	6.51	1.71	1 (very difficult to talk) – 8 (very easy)	4.48%
	Family support	20.90	7.84	1 (very strongly disagree) – 28 (very strongly agree)	4.63%
	Family meal	3.92	1.12	1 (never) – 5 (every day)	1.68%
	Peer support	20.11	7.76	1 (very strongly agree) – 28 (very strongly disagree)	4.03%
	Been bullied	1.63	1.06	1 (not been bullied) – 5 (bullied several times a week)	2.40%
	Online contact	14.19	4.31	1 (almost never) – 20 (almost all the time through the day)	6.21%
	Pref. online comms	7.81	3.55	1 (strongly disagree) – 15 (strongly agree)	8.76%
School factors	Teacher support	11.67	2.73	1 (strongly disagree) – 15 (strongly agree)	1.42%
	Like school	2.93	.86	1 (do not like school at all) – 4 (like school a lot)	1.36%
	School pressure	2.41	.98	1 (not at all) – 4 (a lot)	1.00%
	Classmate support	10.90	2.30	1 (strongly disagree) – 15 (strongly agree)	1.87%

#### Outcome variable

We used a composite measure of mental health from the HBSC Symptom Checklist (HBSC‐SCL). The HBSC‐SCL is a non‐clinical measure containing eight health complaint items. The HBSC‐SCL is bi‐factorial, with one factor representing somatic health problems (e.g., headache), and one representing mental health complaints (feeling low, feeling irritable, sleep difficulties and nervousness; Dey et al., 2015; Hetland et al., 2002). We made use of the subscale representing mental health complaints, which is commonly used trans‐nationally in studies reporting on HBSC data (Ravens‐Sieberer et al., 2008). Responses range from 1 (‘about every day’) to 5 (‘rarely or never’); therefore, higher scores reflect better mental health. These items demonstrated good internal consistency in our data (Cronbach's *α* = .77).

#### Individual‐level variables

##### Loneliness

Loneliness was measured by a single item asking adolescents to rate ‘how often do you feel left out of things?’. This was measured on a 5‐point scale, with higher scores representing greater feelings of being left out (*1* = *never*, *5* = *always*). While previous waves of HBSC conducted in Scotland have used a single‐item, direct measure of loneliness (i.e., ‘Thinking about the last week, how often have you felt lonely?’; Currie et al., 2015), this was not included in the 2018 wave. Feeling left out is, however, an indirect indicator of loneliness and is included in the UCLA Loneliness Scale (Russell, 1996), which is frequently used in epidemiological surveys, and has good psychometric properties (Elphinstone, 2018). Furthermore, direct measures of loneliness may be prone to under‐reporting due to the stigmatized nature of loneliness (Shiovitz‐Ezra & Ayalon, 2012). Therefore, indirect measures may be well suited to adolescent samples where social norms, especially favour connectedness (Barreto et al., 2022; Pitman et al., 2018).

##### Demographic variables

The study controlled for demographic factors including age at the time of survey completion, gender (*female* = *2*, *or male* = *1*) and socioeconomic status (SES). SES was assessed via the use of the Family Affluence Scale (FAS‐III), which is a six‐item assessment of material assets or activities (Torsheim et al., 2016). Items refer to: having your own room; number of cars in the family, holidays abroad; number of computers at home; how many bedrooms in the home; and whether there is a dishwasher at home. Higher scores for each item represent greater affluence. Responses were sum‐scored to obtain an overall FAS‐III score as per HBSC guidance (Torsheim, 2019). We did not have access to school‐level indicators of SES; however, subjective measures of SES are more sensitive indicators among adolescents (Svedberg et al., 2016; Euteneuer, 2014), and individual‐level measures of SES are more strongly associated with youth health outcomes than school or area‐level measures (Pardo‐Crespo et al., 2013).

#### Social‐relationship variables

Social‐relationship variables were created based on available data and HBSC study protocols.

##### Family variables

To examine the influence of family relationship quality, we included measures of family communication and family support. Family support was a composite variable composed of four items (‘I can talk about my problems with my family’, ‘I get the emotional help and support I need from my family’, ‘My family is willing to help me make decisions’, ‘My family really tries to help me’) all rated on a 7‐point scale, with higher scores representing greater perceived family support. This is from the family subscale of the Multidimensional Scale of Perceived Social Support (MSPSS: Zimet et al., 1988), which is well‐validated and used within a range of epidemiological studies (Dahlem et al., 1991). This demonstrated excellent internal consistency (*α* = .96).

Family communication was a composite of two items: ‘how easy is it to talk to your mother about the things that matter?’ and ‘how easy is it to talk to your father about the things that matter?’ which we coded so that responses ranged from 1 (*very difficult*) to 4 (*very easy*), meaning that higher scores represented greater ease of talking with parents. This demonstrated good internal consistency (*α* = .71).

Finally, in relation to family factors, we included an item examining how often adolescents ate a family meal. Following reverse coding, this ranged from 1 (*never*) to 5 (*every day*).

##### Peers and social media

We controlled for several factors relating to peer relationships and social media use. Firstly, a composite variable was created to assess peer support. This included four items (‘My friends really try to help me’; ‘I can count on my friends when things go wrong’; ‘I have friends with whom I can share my joys and sorrows’; ‘I can talk about my problems with my friends’), all scored on a 7‐point scale, with higher scores representing greater support from friends. This composite had excellent internal consistency (Cronbach's *α* = .96). These items represent the ‘friend’ subscale of the MSPSS (Zimet et al., 1988).

We also included an item assessing how often adolescents had been bullied at school, with responses ranging from 1 (I *have not been bullied*) to 5 (*several times a week*).

In relation to social media, we created two composite variables: frequency of online contact and preference for online social interaction. The online contact composite consisted of four items assessing how often adolescents have online contact with: ‘Close friend(s)’; ‘Friends from a larger friend group’; ‘Friends that you got to know through the internet but you didn't know before’; ‘Other people than friends’. This was coded so that responses ranged from 1 (*never or almost never*) to 5 (*almost all the time throughout the day*; *α* = .73).

To assess preference for online social interaction, we used three items (‘On the internet: I talk more easily about secrets than in a face‐to face encounter’; ‘I talk more easily about my inner feelings than in a face‐to‐face encounter’; ‘I talk more easily about my concerns than in a face‐to‐face encounter’) to create a composite variable. Each item was rated from 1 (*strongly disagree*) to 5 (*strongly agree*); therefore, higher scores represented increased preference for online communication (*α* = .92).

#### School community variables

We controlled for several school‐based factors to explore the impact of school (framed as an adolescent's community) on mental health. First, we created a composite variable to investigate perceived teacher support. This included three items (‘I feel that; my teachers accept me as I am’; ‘my teachers care about me as a person’; ‘I feel a lot of trust in my teachers’), which were rated from 1 (*strongly agree*) to 5 (*strongly disagree*). Items were reverse coded so that higher scores reflected higher support from teachers (*α* = .89).

Secondly, we created a composite variable to measure classmate support. This included three items (‘The pupils in my class(es) enjoy being together’; ‘Most of the pupils in my class(es) are kind and helpful’; ‘Other pupils accept me as I am’), which were rated from 1 (*strongly agree*) to 5 (*strongly disagree*). Items were reverse coded so that higher scores reflected higher support from classmates and summed (*α* = .76).

Finally, we included a single‐item measure assessing the extent to which adolescents like school from 1 (*I like it a lot*) to 4 (*I don't like it at all*), which was reverse coded so that higher scores reflected more enjoyment of school. A single‐item measure assessing adolescents' perceived school pressure ranging from 1 (*feel no pressure*) to 4 (*feel a lot of pressure*) was also included.

#### Health and well‐being

Finally, we included two single‐item measures of health and well‐being in our analyses. Self‐rated health was assessed on a four‐point scale, with higher scores representing better health. Overall life satisfaction was scored from 1 to 10, with higher scores representing greater life satisfaction.

#### School attended

To account for the nested structure of the data (i.e., students in schools) and therefore examine school‐level variation in mental health, we used a ‘school ID’ variable. In total, 208 schools were included for analysis, with the number of participating adolescents at each school ranging from 4 to 81, with an average of 30.42 students per school. In total, 234 adolescents sampled (4.4.%) attended independent or fee‐paying schools (8 schools of 208 in the sample).

### Statistical analyses

To answer each of our research questions, we tested a series of multilevel models, which nested students within their schools. Multilevel analyses are commonly used in health‐related research, as they account for variation in outcomes (i.e., mental health) at both the individual and school level (Merlo et al., 2006). Therefore, multilevel modelling allowed us to determine any variation in mental health and its association with loneliness attributable to differences at the school level.

We first tested a null model to determine whether there were significant between‐school differences in mental health and hence, whether multilevel modelling would be an appropriate statistical approach. A likelihood‐ratio test comparing a single‐level regression model to a random intercept multilevel model demonstrated significant clustering at the school level, thus indicating the need to proceed with multilevel modelling.

We then ran a series of multilevel models that incorporated demographic variables as predictors of mental health: individual factors (Model 2), social relationships (Model 3) and finally school factors (Model 4), while accounting for individual school attended. Lastly, we tested a random‐slope model to determine whether the association between loneliness and mental health differed across schools.

All analyses were conducted in RStudio Version 1.3.1093. Multilevel analyses were conducted using the R2MLwiN package (Zhang et al., 2016). A complete case analysis was conducted on each model, with missing cases subject to listwise deletion, and missing data were not imputed. This resulted in a sample size of 3897 in our final model.

## RESULTS

### Descriptive statistics

Table 1 displays descriptive statistics for our sample. Of our sample, 51.35% were female. The mean score for loneliness was mid‐range (mean = 2.4, range = 1–5) representing a score which falls between feeling left out ‘sometimes’ and ‘hardly ever’. About 12% of the sample reported feeling left out often or always. We present bivariate correlations between variables in Table 2.

**TABLE 2:** Bivariate correlations of key variables

	1	2	3	4	5	6	7	8	9	10	11	12	13	14	15	16	17	18
1. Mental Health																		
2. Left out	−.471**																	
3. Gender	−.133**	.128**																
4. Age	−.234**	.066**	−.017															
5. SES	.94**	−.078**	.000	.022														
6. Health	.310**	−.213**	−.009	−.167**	.148**													
7. Life satisfaction	.502**	−.378**	−.021	−.251**	.133**	.421**												
8. Family communication	.338**	−.246**	.021	−.193**	.072**	.214**	.353**											
9. Family support	.199**	−.109**	.010	−.185**	.070**	.134**	.238**	.228**										
10. Family meal	.197**	−.149**	.009	.040**	.139**	.187**	.237**	.191**	.098**									
11. Peer support	.116**	−.133**	.116**	−.105**	.043**	.104**	.146**	.084**	.726**	.037**								
12. Been bullied	−.280**	.391**	.024	−.045**	−.047**	−.080**	−.214**	−.155**	−.051**	−.075**	−.086**							
13. Online contact	−.080**	−.070**	.060**	.233**	.094**	−.039**	−.038**	.046**	−.006**	−.012	.094**	−.031*						
14. Pref. online comms	−.249**	.154**	−.024	.264**	−.032*	−.139**	−.220**	−.200**	−.119**	−.113**	−.042**	.102**	.305**					
15. Teacher support	.352**	−.220**	.022	−.309**	.029*	.223**	.353**	.294**	.169**	.163**	.112**	−.135**	−.120**	−.222**				
16. Like school	.369**	−.232**	.038**	−.246**	.075**	.243**	.376**	.221**	.160**	.179**	.120**	−.165**	−.125**	−.223**	.496**			
17. School pressure	−.465**	.294**	.078**	.380**	−.010	−.204**	−.334**	−.224**	−.159**	−.110**	−.104**	.151**	.111**	.203**	−.346**	−.365**		
18. Classmate support	.392**	−.389**	−.081**	−.233**	.082**	.261**	.373**	.280**	.135**	.161**	.140**	−.361**	.012	−.169**	.477**	.384**	−.302**	

*Note*: *p < .05, **p < .01.

### Multilevel analysis

Table 3 displays the results from our multilevel models. Results from the likelihood‐ratio test comparing the null multilevel model to a single‐level model demonstrated significant variation in adolescent mental health across schools, *χ* ^2^(1) = 172.34, *p* < .001. An intraclass coefficient (ICC) of .082 was recorded for the null model, indicating that 8.2% of the variation in mental health was attributable to between‐school differences.

**TABLE 3:** Results of multilevel models

Outcome variable: Mental health					
Domain	(1)	(2)	(3)	(4)	Random slopes model for loneliness
Fixed effects					
Intercept	19.862*** (0115)	16.327*** (.625)	14.428*** (.733)	11.956*** (.805)	11.653*** (.801)
Individual/demographics					
Left out	−2.158*** (.056)	−1.488*** (.058)	−1.166*** (.068)	−.933*** (.067)	−.874*** (.067)
Gender		−.721*** (.099)	−.834*** (.108)	−.731*** (.104)	−.719*** (.103)
Age		−.314*** (.031)	−.206*** (.035)	.033 (.036)	.036 (.036)
SES		.035 (.022)	.016 (.023)	.025 (.022)	.029 (.022)
Health		.569*** (.082)	.457*** (.089)	.348*** (.086)	.339*** (.086)
Life satisfaction		.688*** (.031)	.610*** (.036)	.491*** (.036)	.500*** (.036)
Social relationships					
Family communication			.266*** (.035)	.212*** (.034)	.197*** (.034)
Family support			.032** (.010)	.028** (.010)	.031** (.010)
Family meal			.162*** (.050)	.115* (.048)	.127** (.048)
Peer support			−.010 (.010)	−.014 (.010)	−.016 (.010)
Been bullied			−.439*** (.055)	−.334*** (.055)	−.358*** (.057)
Online contact			−.053*** (.014)	−.039** (.014)	−.040** (.014)
Pref. online comms			−.080*** (.016)	−.058*** (.016)	−.057*** (.016)
School /community					
Teacher support				.073** (.024)	.071** (.024)
Like school				.283*** (.074)	.283*** (.073)
School pressure				−1.014*** (.061)	−1.001*** (.061)
Classmate support				.055 (.029)	.063* (.039)
School intercept variance	14.155 (.278)	11.433 (.234)	10.783 (.241)	9.728 (.220)	6.933 (1.1923)
School slope variance (loneliness)					.282 (.223)
Covariance					.197 (.535)
Observations	5155	4756	3975	3897	3897
AIC	28,296.38	25,100.79	20,768.18	19,967.63	19,919.94

*Note*: *p < .05, **p < .01, ***p < .001.

Models 1–4 show the results from our sequential model building, where we tested demographic, social and school factors independently. Model 4 serves as our final model, where social‐ecological domains are tested together, controlling for school‐level clustering. Results from the final model demonstrated that mental health problems were reported more frequently among those who felt greater loneliness (*b* = −.93, *SE* = .07) and among female adolescents (*b* = −.73, *SE* = .10). Less frequent mental health problems were reported by those with higher self‐rated physical health (*b* = .35, *SE* = .09) and greater life satisfaction (*b* = .49, *SE* = .04). Age, though associated with mental health in preceding models, was not significant when controlling for school‐related factors. Family affluence was not associated with mental health.

Several variables relating to social relationships were associated with better mental health: family communication (*b* = .21, *SE* = .03), increased family support (*b* = .03, *SE* = .01) and more frequently eating a family meal together (*b* = .12, *SE* = .05). Conversely, social‐relationship factors that were associated with more frequent mental health problems included being bullied in school more frequently (*b* = −.33, *SE* = .06), more frequently using social media to contact friends and family (*b* = .04, *SE* = .01) and an increased preference for online communication (*b* = −.06, *SE* = .02).

Multiple variables relating to school were predictive of mental health. Perceived teacher support (*b* = .07, *SE* = .02) and a greater enjoyment of school (*b* = .28, *SE* = .07) were associated with less frequent mental health problems. Alternatively, those who reported experiencing greater school‐related pressure reported more frequent mental health problems (*b* = −1.01, *SE* = .06).

Finally, we tested a random slopes model to determine whether the effect of loneliness on mental health differed depending on the school attended. Results demonstrated that this effect did differ across schools. Specifically, the negative effect of loneliness on mental health was stronger in schools with lower average mental health scores. In addition, the between‐school variation in mental health was greater among more lonely young people.

As a supplemental check on our models, we tested a set of cross‐level interactions between the key school factors (i.e., teacher relationship, the extent that adolescents like school, school pressure and classmate support) and loneliness, to see whether this accounted for some of the variation between schools in the effect of loneliness on mental health. No significant interactions were found.

## DISCUSSION

In our sample, about 12% of adolescents reported feeling left out often or always, which is representative of prevalence rates of adolescent loneliness reported elsewhere (Office for National Statistics, 2018b; Yang et al., 2020), where 11% of 10‐ to 15‐year‐olds in the United Kingdom and 9% of 14‐year‐olds in England felt lonely often. We also found that schools accounted for a proportion of the variation between adolescents in mental health. Our study further advances the evidence base by demonstrating that the negative association between loneliness and mental health is greater in schools with lower average mental health scores, and that between‐school differences in mental health were greater among more lonely adolescents. These findings suggest that (a) loneliness may be especially detrimental in schools where the mental health of students is poorer, and (b) among adolescents reporting loneliness, the school they attend is more predictive of their mental health than for their less lonely peers.

### Social‐ecological correlates of adolescent mental health

Across all our models, loneliness was strongly, negatively associated with mental health. In practical terms, when controlling for social‐ecological predictors and variation in mental health at the school level, each one‐point increase in loneliness was associated with a decrease of .93 on the mental health scale. This was expected given the noted links between loneliness and poorer mental health and well‐being in adolescence (Goodfellow et al., 2022; Loades et al., 2020).

We also demonstrated that higher ratings of self‐rated physical health and life satisfaction were associated with better mental health. Again, there are strong associations between good physical and mental health (Aarons et al., 2008), and life satisfaction and improved mental health in adolescence (Proctor et al., 2009). Thus, these associations were expected.

In terms of social‐relationship factors, we found that a range of family factors were associated with better mental health of adolescents. Specifically, improved family communication, greater perceived support from family and eating a family meal together more frequently were associated with better mental health. In contrast to this, we found that peer support was not associated with mental health.

Previous research has demonstrated that the role of peer and parental connections may have differing impacts on mental health, with insecure parental attachment (though not peer attachment) being associated with externalizing and emotional disorders among adolescents (Oldfield et al., 2016). Eating a family meal together has also been demonstrated to increase well‐being among adolescents (Elgar et al., 2013). Therefore, despite adolescence being marked by a transition from family to peers as a primary source of socializiation (Goossens, 2018; Laursen & Hartl, 2013), a supportive family environment appears to be key to sustaining good mental health.

We noted a strong negative association between increased frequency of being bullied and mental health. This was unsurprising given the large body of evidence documenting the negative impact of bullying on mental health in adolescence (Jadambaa et al., 2019; Moore et al., 2017). However, it is important to note that in our study, we measured bullying specifically within a school context, and we did not include the mental health impacts of bullying outside of a school setting. Bullying from siblings can increase loneliness (Yang et al., 2020), while teachers' dismissal of their students' loneliness experiences is detrimental to mental health, (Verity et al., 2022). Future research should explore how relationships with school staff and siblings impact loneliness and mental health, and their prospective relationship.

Our study adds to the discourse relating to social media use and mental health in adolescence. We found that both increased frequency of online contact with friends and others and a preference for online communication were associated with poorer mental health. Social media use is highly prevalent among adolescents, with over a third of adolescents across 29 countries reporting intense social media use (Boer et al., 2020). However, evidence on the relationship between the frequency of social media use and mental health is mixed and complicated by the use of differing measures and definitions. Some research has found greater time spent using social media is associated with poorer body image, poorer sleep and increased depressive symptoms, particularly among girls (Kelly et al., 2018), while other research has found that frequent social media use is most detrimental to life satisfaction among younger adolescents (Orben et al., 2022). However, while there is a notable negative effect of social media use on mental health, these effects can be small, and directionality unclear (Orben, 2020). Negative online social interactions have also been associated with increased adolescent loneliness (Magis‐Weinberg et al., 2021), suggesting that it may be important to consider the quality of online interactions on adolescents' mental health, rather than simply the amount of time spent online.

Finally, several school factors were associated with mental health. As expected, mental health was higher among adolescents who perceived greater teacher support, and those who reported liking school, and lower among those who experienced greater school pressure. This resonates with other findings indicating that improved school climate, including supportive teacher relationships, and increased sense of belonging, can bolster adolescent mental health (Singla et al., 2021), and the evidence that an over‐emphasis on academic achievement may be detrimental to mental health (Högberg et al., 2020; László et al., 2019).

Interestingly, while teacher support was associated with better mental health, classmate support was not. Adolescents may receive simultaneous social support from multiple interpersonal relationships (Kenny et al., 2002), and these distinct sources of social support may be differently related to psychosocial outcomes (Guan & Fuligni, 2016). There is evidence that peers and adults can provide different forms of support to adolescents, but what is of importance is the quality of these relationships, rather than the quantity of connections (Melton et al., 2021). Findings from our study reinforce the importance of positive student–teacher relationships for positive mental health. Combined with our finding that family support, but not peer support, was associated with improved adolescent mental health, our results suggest that supportive adults, across a range of social settings, may be of key importance in supporting the mental health of adolescents.

### Between‐school variation in mental health and differing effects of loneliness

Using multilevel modelling techniques, our study found that the school an adolescent attended accounted for an initial 8% of the variation in mental health among our sample, although this number decreased when adjusted for covariates. Additionally, our results showed that the effect of loneliness on mental health differed across schools. This means that, in some schools, loneliness was more important for mental health than in others. Specifically, the negative effect of loneliness on mental health was stronger in schools with lower than average mental health scores. In addition, between‐school differences in mental health were greater among adolescents with high levels of loneliness. Thus, our results indicate that there are important school‐based factors that affect the mental health of lonely adolescents and that it may be particularly vital to tackle loneliness in schools where mental health is already likely to be poor.

Overall, our findings suggest that tackling loneliness could be a crucial lever to improving the mental health of adolescents. Research demonstrates that risk factors for adolescent loneliness include, though are not limited to: experiencing social transitions, for example, moving from primary to secondary school (Siva, 2020; Sundqvist & Hemberg, 2021); low socioeconomic position (Madsen et al., 2019; Varga et al., 2014); having a disability (Maes et al., 2017; Office for National Statistics, 2019); or being of minority sexual orientation (Gorczynski & Fasoli, 2021; Marquez et al., 2022). Therefore, it may be particularly critical to develop interventions to tackle loneliness and foster meaningful social connections among these groups.

Furthermore, our study highlights that while loneliness is consistently detrimental to adolescent mental health, among the adolescents who are the most lonely, the school they attend can provide an important buffer for their mental health. This underscores the importance of developing school‐based interventions to reduce loneliness. These are likely to be key to improving mental health among adolescents, but are likely to be particularly effective among the loneliest young people.

Finally, based on findings in this study, supportive teachers are important for adolescent mental health. It may therefore be important to involve teachers in the delivery of interventions aiming to improve loneliness. Indeed, young people themselves have suggested that loneliness be incorporated into teacher's mental health first‐aid training to support teaching staff to identify loneliness, and how to meaningfully engage to reduce its negative effects (Mental Health Foundation, 2021).

### Limitations

This study has a number of limitations. First, the data were cross‐sectional and therefore limit our ability to make causal inferences. Due to the cross‐sectional nature of the data, we also cannot rule out bi‐directionality of associations between mental health and loneliness. However, our analyses uncovered new knowledge regarding the association between loneliness and mental health, which can inform future longitudinal studies.

Second, we used the most contemporary wave of HBSC data in order to maximize the public health relevance of our findings. However, this wave of HBSC did not include the optional item referring directly to loneliness, but rather, asked how often adolescents had felt ‘left out’. Nevertheless, direct measures of loneliness are highly correlated with indirect measures contained within the UCLA Loneliness Scale (Russell, 1996; Russell et al., 1980). This scale includes a measure of feeling left out, indicating that it is a valid and appropriate proxy for loneliness (Eccles et al., 2020).

Finally, a key limitation of the current study is that while we were able to identify that mental health showed significant variation between schools, and that the relationship between loneliness and mental health varied, we were unable to identify which school‐level factors were associated with these differences. However, given the insignificant cross‐level interactions between‐school factors (e.g., teacher support, extent to which adolescents like school) and loneliness, we know that the variability across schools in the relationship between loneliness and mental health was not related to these variables. Due to data availability, we did not have school‐specific contextual data (e.g., measures of deprivation) to explain these differences in mental health. However, a recent randomized controlled trial found that school‐level factors such as urban location, higher percentage of free school meals, and a higher percentage of White British students were associated with poorer pupil mental health (Ford et al., 2021). These factors may, therefore, be useful in identifying schools where mental health is lower than average. Future research should aim to further integrate data on the wider contextual environment of schools to more fully understand the drivers of school‐level variability in mental health, and school‐level factors that protect lonely young people from worsening mental health.

## CONCLUSIONS

Our results demonstrate that loneliness is strongly associated with poorer mental health in adolescence. Additionally, while previous studies have investigated school‐level variation in mental health, our study advances the evidence by not only confirming between‐school differences in mental health, but also demonstrating that the association between loneliness and mental health varied between schools. Importantly, our results show that the between‐school variation in mental health was greater among more lonely adolescents, and that the negative relationship between loneliness and mental health was strongest in schools where mental health was below average. Therefore, among adolescents experiencing loneliness, the school they attend is a key contributing factor to their mental health. Public health approaches should promote whole‐school interventions which aim to support adolescent mental health, especially for young people experiencing higher levels of loneliness, or develop interventions to reduce loneliness in schools where mental health is already likely to be low.

## AUTHOR CONTRIBUTIONS

**Claire Goodfellow:** Conceptualization; data curation; formal analysis; writing – original draft; writing – review and editing. **Malachi Willis:** Conceptualization; data curation; formal analysis; writing – original draft; writing – review and editing. **Joanna Inchley:** Conceptualization; writing – original draft; writing – review and editing. **Kalpa Kharicha:** Conceptualization; writing – original draft; writing – review and editing. **Alastair H. Leyland:** Conceptualization; writing – original draft; writing – review and editing. **Pamela Qualter:** Conceptualization; writing – original draft; writing – review and editing. **Sharon Simpson:** Conceptualization; writing – original draft; writing – review and editing. **Emily Long:** Conceptualization; funding acquisition; writing – original draft; writing – review and editing.

## FUNDING INFORMATION

This work was supported by the Economic and Social Research Council [ES/T008679/1], the Medical Research Council [MC_UU_00022/1, MC_UU_00022/2, MC_UU_00022/3] and the Chief Scientist Office [SPHSU16, SPHSU17, SPHSU18]. EL is also supported by MRC Skills Development Fellowship Award [MR/S015078/1].

## CONFLICT OF INTEREST

The authors declare that they have no conflict of interest.

### OPEN RESEARCH BADGES

This article has earned an Open Data badge for making publicly available the digitally‐shareable data necessary to reproduce the reported results. The data is available at http://www.hbsc.org/.

## ETHICAL APPROVAL

All participating countries within the HBSC network adhere to ethical guidelines and principles as described in the HBSC study protocols. The HBSC study is conducted according to the guidelines of the Declaration of Helsinki. Ethical approval for the HBSC 2018 survey in Scotland was obtained from the University of St Andrews School of Medicine Ethics Committee.

## CONSENT TO PARTICIPATE

Informed consent was given by school administrators (explicit) and parents (implicit) prior to survey administration. Pupils actively consented by participating in the survey. They were informed that participation was voluntary and confidential, and they could skip any questions that they did not want to answer.

## CONSENT FOR PUBLICATION

No identifying information about participants is available in the article.

## Data Availability

Access to the survey data is restricted to HBSC research teams for a period of 3 years from survey completion. After this time, the data are available for external use by agreement with the International Coordinator and Principal Investigators. The HBSC mandatory 2017/2018 data set for Scotland (used in this study) will be made publicly accessible 3 years after survey completion. HBSC datasets are available via the Health Behaviour in School‐aged Children repository at http://www.hbsc.org/.
